# Effect of visceral fat on onset of metabolic syndrome

**DOI:** 10.1038/s41598-025-01389-1

**Published:** 2025-05-30

**Authors:** Hiroto Bushita, Naoki Ozato, Kenta Mori, Hiromitsu Kawada, Yoshihisa Katsuragi, Noriko Osaki, Tatsuya Mikami, Ken Itoh, Koichi Murashita, Shigeyuki Nakaji, Yoshinori Tamada

**Affiliations:** 1 Department of Medical Data Intelligence, Research Center for Health-Medical Data Science, Hirosaki University Graduate School of Medicine, Aomori, Japan; 2 Human Health Care Products Research Laboratories, Kao Corporation, Tokyo, Japan; 3 Department of Active Life Promotion Sciences, Hirosaki University Graduate School of Medicine, Aomori, Japan; 4 Research and Development, Kao Corporation, Tokyo, Japan; 5 Department of Preemptive Medicine, Innovation Center for Health Promotion, Hirosaki University Graduate School of Medicine, Aomori, Japan; 6 Department of Stress Response Science, Biomedical Research Center, Hirosaki University Graduate School of Medicine, Aomori, Japan; 7 Research Institute of Health Innovation, Hirosaki University, Aomori, Japan; 8 Department of Social Medicine, Hirosaki University Graduate School of Medicine, Aomori, Japan

**Keywords:** Metabolic syndrome (MetS), Visceral fat, Onset prediction models, SHapley additive exPlanations (SHAP), Machine learning, Early detection, Computational biology and bioinformatics, Health care, Medical research, Mathematics and computing

## Abstract

This study analysed the effects of visceral fat on metabolic syndrome (MetS) and developed an algorithm to predict its onset using health examination data from the Iwaki Health Promotion Project in Japan. The dataset included 213 cases of MetS onset within three years and 1320 non-onset cases. The data was split into training and test sets with an 8:2 ratio. In the training set, the MetS onset group had significantly higher visceral fat area than the non-onset group (p < 0.00001). A cut-off value of 82 cm2 for the visceral fat area was determined, with an AUC of 0.86. Additionally, a machine learning algorithm utilizing seven non-invasive factors, including visceral fat, achieved high accuracy with a five-fold cross-validation AUC of 0.90 in the training set and 0.88 in the test set. Visceral fat was identified as the main factor, as supported by the SHAP value. In conclusion, this study found visceral fat to be crucial in predicting the onset of MetS, and a high-accuracy onset prediction algorithm based on non-invasive parameters, including visceral fat, was developed.

## Introduction

Cardiometabolic diseases, including metabolic syndrome (MetS), have become the leading cause of death worldwide in the past 20 years^1^. MetS involves several risk factors such as abdominal obesity, hyperglycaemia, hypertension, and dyslipidaemia and is becoming a serious problem worldwide^2–4^. In 2020, approximately 25.8 million children and 35.5 million adolescents worldwide were affected by MetS^5^. Therefore, the prevention and mitigation of MetS is an urgent issue. Various preventive approaches have been proposed (exercise guidance^6^ and dietary guidance^7–9^). On the other hand, it has been reported that behavioural changes can be expected by knowing the risk of disease^10^. Therefore, the ability to predict the onset of MetS years in advance is considered to be one of the most useful tools for preventing the development of MetS. In previous studies, the severity of current MetS has been estimated using volume data from body scanners and demographic data (age, sex, BMI, etc.)^11^. In addition, several algorithms for predicting the onset of MetS have been developed^12–23^. However, the definition of MetS differs in different countries^24^. In Japan, visceral fat accumulation is essential for diagnosis, with a cut-off value of 100 cm^2^^25,26^. One reason for this is that Asians are more susceptible to MetS than Westerners, and even those with a low BMI accumulate visceral fat^27,28^. Thus, accumulating research knowledge in accordance with each country’s standards is important.

The relationship between visceral fat and MetS has been the subject of several cross-sectional studies in Japan, China, South Korea, and the United States^29–34^.

A 3.3-year longitudinal study in the United States^35^ and a 17-month longitudinal study in South Korea^36^ reported that visceral fat increases MetS onset risk as defined by the waist size. A 3-year longitudinal study in Japan reported that an increase in visceral fat is correlated with MetS risk factors^37^. However, no large-scale studies have been conducted in Japan measuring longitudinal changes in visceral fat area (VFA) in the same subjects over a period of 1–6 years. Moreover, the relationship between MetS onset risk and visceral fat remains relatively unexplored.

The definition of metabolic syndrome includes visceral fat, hyperglycaemia, hypertension and lipid disorders. Direct measurement of visceral fat is difficult due to exposure and cost considerations. Therefore, abdominal circumference measurement has been used as an alternative to visceral fat measurement, but previous studies have shown only a weak correlation between VFA and waist circumference (men: r = 0.68, women: r = 0.65) and considerable individual-level variation between VFA and waist circumference measurements^26^. Therefore, simplified models using abdominal circumference have been constructed in previous literature^15,16,19,21^. On the other hand, direct measurement of visceral fat is of course preferable when constructing predictive models of MetS. We have developed a medical device capable of non-invasively measuring visceral fat directly^58^ and have been collecting health data including visceral fat since 2015. This study used the data for the period 2015–2020 to clarify the relationship between visceral fat and MetS onset. An algorithm to predict MetS onset was developed.

## Results

### Physical characteristics

**Fig. 1:**
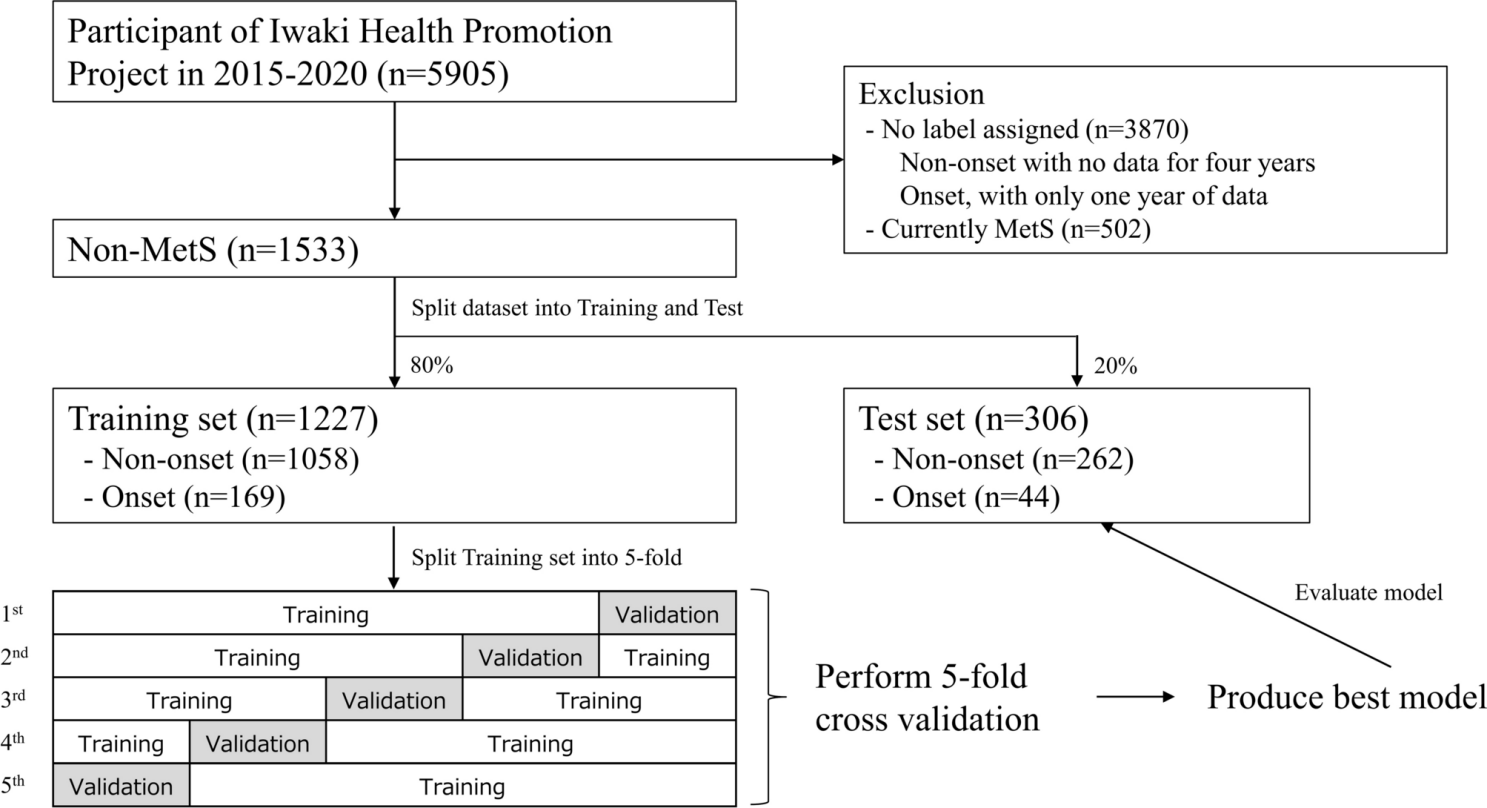
Research steps employed in this study.

In the data for cross validation (*n* = 1227), the onset and non-onset groups comprised 169 and 1058 cases, respectively. In the test data (*n* = 306), the onset and non-onset groups comprised 44 and 262 cases, respectively (Fig. 1). The MetS incidence in each dataset was 13.8% and 14.4%, respectively. Table 1 shows the subject background information at the baseline in the cross-validation data.

**Table 1:** Physical characteristics at baseline for the MetS onset and non-onset groups. The p-value indicates the difference between the MetS onset and non-onset groups. (a) Mann–Whitney U test (corrected for ties) was used for continuous variables; (b) Fisher’s exact test was used for categorical variables.

	Non-onset	Onset	P-value
Number [n]	1058	169	
Gender: Female / Male [n] (%)	710/348 (67.1/32.9)	62/107 (36.7/63.3)	< 0.001^b^
Age [years] (median [IQR])	52.00 [38.75, 62.00]	57.00 [45.00, 66.00]	< 0.001^a^
Height [cm] (median [IQR])	159.40 [154.80, 166.80]	162.50 [157.22, 171.73]	< 0.001^a^
Body mass index [kg/m2] (median [IQR])	21.80 [19.90, 23.50]	24.55 [23.40, 26.60]	< 0.001^a^
Waist circumference [cm] (median [IQR])	81.70 [76.90, 86.60]	90.20 [86.80, 95.65]	< 0.001^a^
Triglycerides [mg/dL] (median [IQR])	72.00 [54.00, 103.00]	110.00 [82.00, 141.75]	< 0.001^a^
HDL-cholesterol[mg/dL] (median [IQR])	67.00 [57.00, 78.00]	56.50 [49.00, 64.00]	< 0.001^a^
LDL-cholesterol [mg/dL] (median [IQR])	114.00 [94.00, 133.50]	123.00 [105.00, 148.00]	< 0.001^a^
Systolic blood pressure [mmHg] (median [IQR])	118.00 [107.00, 128.00]	131.00 [120.00, 143.75]	< 0.001^a^
Diastolic blood pressure [mmHg] (median [IQR])	71.00 [64.00, 79.00]	81.00 [73.25, 89.75]	< 0.001^a^
Fasting plasma glucose [mg/dL] (median [IQR])	84.00 [78.00, 91.00]	93.00 [86.00, 100.75]	< 0.001^a^
HbA1c [%] (median [IQR])	5.60 [5.40, 5.80]	5.80 [5.50, 6.00]	< 0.001^a^
Energy intake [kcal/day] (median [IQR])	1737.63 [1429.92, 2160.99]	1945.28 [1660.52, 2276.05]	< 0.001^a^
Number of drinking days [day/week] (median [IQR])	0.00 [0.00, 0.00]	0.00 [0.00, 0.00]	0.654^a^
Alcohol intake(energy-adjusted) [g/day/kcal] (median [IQR])	0.00 [0.00, 0.01]	0.00 [0.00, 0.02]	0.003^a^
Number of cigarettes smoked [n/day] (median [IQR])	0.00 [0.00, 10.00]	0.00 [0.00, 15.00]	0.008^a^
Exercise intensity [METs・hour/week] (median [IQR])	0.00 [0.00, 3.50]	0.00 [0.00, 7.00]	0.399^a^
Sleeping time [min./day] (median [IQR])	420.00 [360.00, 450.00]	420.00 [390.00, 480.00]	0.003^a^

In the onset group, 36.7% were females and 63.3% were males, and in the non-onset group, 67.1% were females and 32.9% were males. Onset and gender were significantly related (p < 0.001). The median age in the onset group was 57 years, which was significantly higher than that in the non-onset group (52 years). Compared with the non-onset group, the onset group had a significantly greater height, and a significantly larger BMI, VFA, and waist circumference (p < 0.001). In addition, triglycerides (TG), high-density lipoprotein cholesterol (HDLC), low-density lipoprotein cholesterol (LDLC), systolic blood pressure (SBP), diastolic blood pressure (DBP), blood sugar (BS), and haemoglobin A1c (HbA1c), which are factors involved in MetS, were significantly higher (p < 0.001) in the onset group. Energy intake (BDHQ), alcohol intake (BDHQ, after adjusting for energy), number of cigarettes smoked, and sleeping time were significantly higher or longer (p < 0.001, p = 0.003, p = 0.008, p = 0.003) in the onset group. No significant differences were observed between the two groups with respect to number of drinking days and exercise intensity (p = 0.654, p = 0.399).

### Effect of visceral fat on MetS onset

**Fig. 2:**
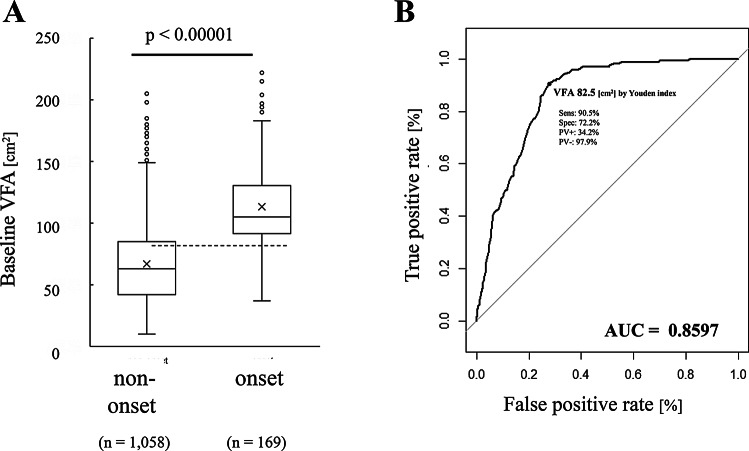
Effect of baseline visceral fat on MetS onset (**a**) VFA values at baseline in the MetS onset group (*n* = 169) and non-onset group (*n* = 1,058), The p value indicates the difference between the MetS onset and non-onset groups using the Mann–Whitney U test (corrected for ties); (**b**) ROC curve of baseline VFA that determines MetS onset risk. The cut-off value was calculated using the Youden index.

The VFA at baseline was compared between the MetS onset and non-onset groups. In the onset group, 45% were VFA < 100 cm^2^ cases and 55% were VFA ≥ 100 cm^2^ cases. In the non-onset group, 86.1% were VFA < 100 cm^2^ cases and 13.9% were VFA ≥ 100 cm^2^ cases. The VFA quartile values in the onset group were as follows: 25%, 92 cm^2^; 50%, 105 cm^2^; and 75%, 130 cm^2^. In the non-onset group, the values were as follows: 25%, 42 cm^2^; 50%, 63 cm^2^; and 75%, 85 cm^2^, with the VFA being significantly higher in the onset group (Fig. 2A; p < 0.001). As gender^38^, age^38^, number of cigarettes smoked^39^, alcohol intake^40^ and exercise^41^ are known to be associated with VFA, logistic regression analysis was performed while adjusting for these as confounding factors, but a significant association was found between VFA and the presence of metabolic syndrome. Logistic regression analysis confirmed that there was no change in these results even after adjusting for gender, age, number of cigarettes smoked, alcohol intake, and exercise intensity (p < 0.001). AUC for the ROC curve was 0.8597 ± 0.0241 (95% Confidence Interval). The cut-off values based on the Youden index and the ROC curve closest to (0,1) were 82.5 cm^2^ (Fig. 2B) and 85.5 cm^2^, respectively. These results suggest that baseline VFA alone can predict the onset of MetS. It was confirmed that 45% of the non-onset group did not experience the onset of MetS despite their baseline VFA exceeding 100 cm^2^.

### Construction and validation of MetS onset prediction model

**Fig. 3:**
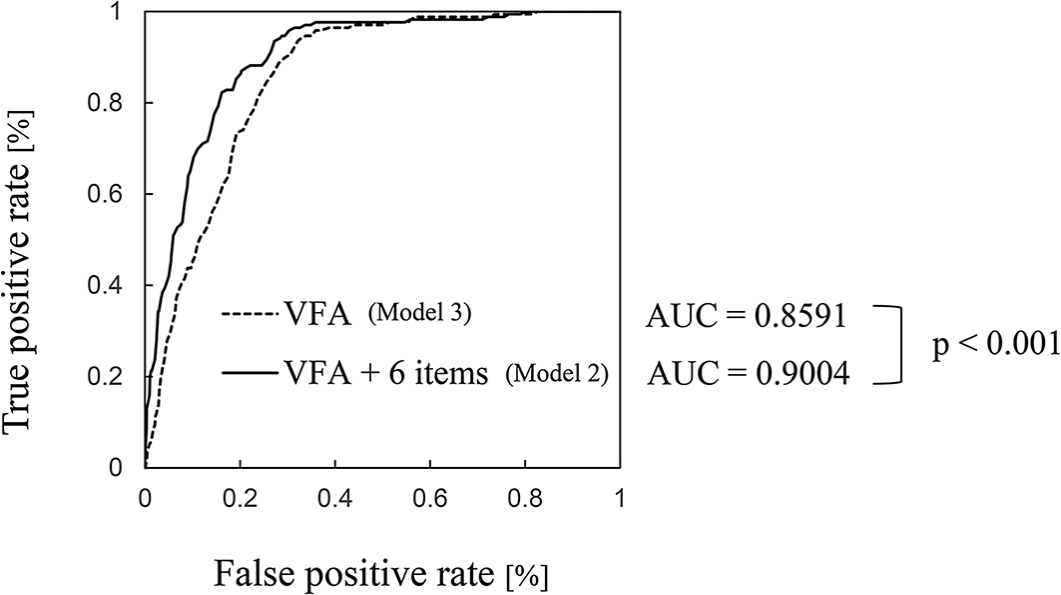
Comparison of AUC during cross-validation of the two models for MetS onset prediction. Model 3 was constructed using Elastic-Net, and the input feature was VFA. Model 2 was constructed using LightGBM, and the input features were VFA, BMI, number of cigarettes smoked, sex, age, SBP, and DBP. The AUC difference was calculated using the DeLong’s test.

The baseline VFA was determined to be an important factor for the prediction of MetS onset; thus, a model for predicting MetS onset was constructed using baseline visceral fat. First, 18 different machine learning models compatible with SHAP were constructed using only baseline VFA as the input parameter, and the prediction model with the highest prediction accuracy was selected. The prediction model constructed using Elastic-Net yielded a cross-validation AUC of 0.8591 ± 0.0364 and a test AUC of 0.8686 ± 0.0696 (95% Confidence Interval) (Fig. 3, Supplemental Table 1). Supplemental Fig. 1 shows the flowchart of model construction and presents the hyperparameters optimised using grid search. The numeric variables were standardized to ensure a consistent scale. For VFA data, there were no missing values. An Elastic-Net classifier was then applied, utilizing a mixing alpha of 5 and optimizing for binomial deviance to construct the predictive model.

A previous study reported on a MetS onset prediction model that was constructed using BMI, number of cigarettes smoked, gender, age, DBP, SBP, and other factors. Previous epidemiological research has suggested that these are important factors ^12,14,42–44^. Therefore, we built an onset prediction model based on visceral fat and seven parameters from the literature^12^. First, we constructed 18 different machine learning models using these eight input parameters, and selected the model with the highest prediction accuracy. Results confirm the accuracy of the model (cross-validation AUC = 0.8992 ± 0.0317, test AUC = 0.8845 ± 0.0660 (95% Confidence Interval)) with no significant difference in AUC difference (Model 1). Next, we ranked the parameters based on their SHAP values. We then constructed 18 models with seven input parameters after excluding the lowest ranked parameter (number of drinking days). We selected the model with the highest prediction accuracy (Model 2). This process was repeated until only one parameter remained (Model 3).

Results showed that Model 2, constructed the seven input features: visceral fat, BMI, number of cigarettes smoked, gender, age, DBP, and SBP, and trained with LightGBM, had the highest prediction accuracy (cross-validation AUC = 0.9004 ± 0.0316, test AUC = 0.8836 ± 0.0662 (95% Confidence Interval)), with no significant differences in AUC differences (Fig. 3, Supplemental Table 1)). The onset and non-onset cases were correctly determined with accuracies of 82% and 84%, respectively, when the Matthews correlation coefficient (MCC)^45^ was maximised (minimising false positives and false negatives). The correct answer rate was 84%. Supplemental Fig. 2 shows the flowchart for model construction and hyperparameter optimisation using grid search. We first preprocessed the numeric variables through data cleansing and standardization (Supplemental Fig. 2.). Missing numeric variables were imputed with their median, and indicator variables were created to mark records with data quality issues. We then applied an Elastic-Net classifier using L2 regularization and binomial deviance, and subsequently used its predictions as input features for a LightGBM model to enhance prediction accuracy. For categorical variables, we employed both one-hot encoding and ordinal encoding, using the transformed data either in conjunction with Elastic-Net and LightGBM or directly with LightGBM for the final predictions. Missing categorical variables were either treated as a new category “missing” in One-Hot Encoding or as a specific value expressing missingness (-2 in this case) in Ordinal encoding. As in Model 3, the missing percentage in the data used for Model 2 was 0.40%.

Replacing VFA with waist circumference in the VFA-only model (Model 3) and the optimised model that included VFA (Model 2) significantly decreased the prediction accuracy (Supplemental Fig. 3 and 4). Therefore, VFA had a greater contribution to the prediction accuracy than waist circumference when predicting MetS onset.

**Fig. 4:**
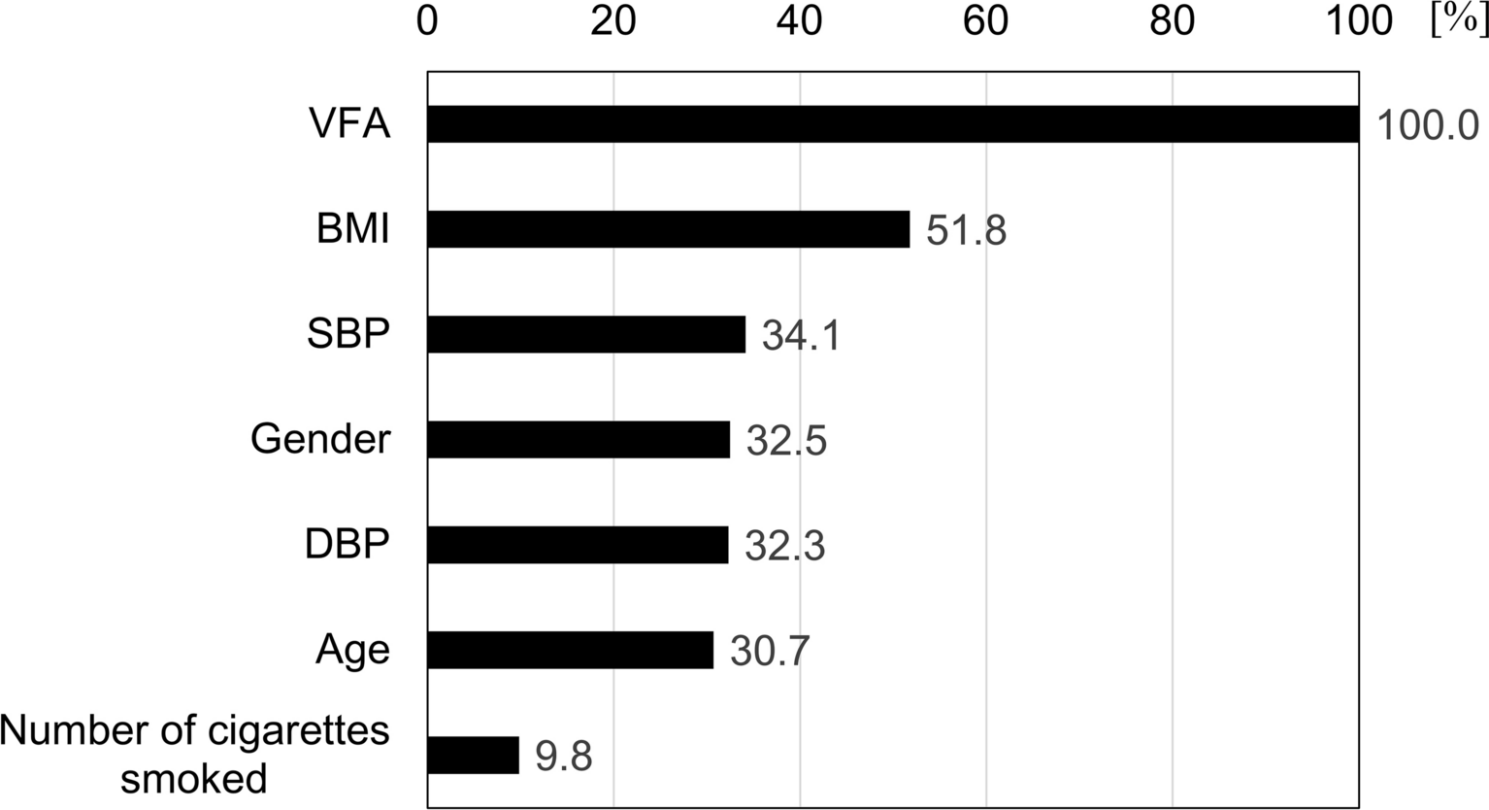
Feature importance based on the SHAP value.

In the optimised MetS onset prediction model (Model 2), the SHAP value of each individual was calculated (Fig. 4) as the feature impact based on the SHAP value. An examination of the influence of each item (gender, age, VFA, BMI, DBP, SBP, and number of cigarettes smoked) on MetS onset showed that VFA was the largest contributor to the prediction of MetS onset. BMI was the second most influential factor, but its feature effect was approximately half that of VFA (Fig. 4).

## Discussion

Although solutions and anti-obesity drugs to improve metabolic syndrome are being developed worldwide, the number of people with metabolic syndrome is increasing every year^2^. As the cost of healthcare increases with metabolic syndrome^46^, the burden on the state increases. In Japan, specific health guidance is actually provided to prevent and improve metabolic syndrome. However, this guidance led to a mild improvement in obesity, but no improvement in blood pressure, blood glucose or lipids, suggesting that the intervention methods of health guidance need to be reviewed in order to improve the health status of the population^47^. In addition, the specific health guidance did not result in any change in healthcare costs^48^. Therefore, we considered that an early monitoring tool is important as one of the methods to prevent metabolic syndrome. One possible way to implement the model developed in this study is to use it on a visceral fat scale. It is expected to be used to motivate health promotion at health check-ups and health events. Challenges to this model include regional characteristics.

Diet and exercise management are important in preventing MetS^49^; however, predicting the onset is also an important approach. In Japan, visceral fat level is an essential parameter in the diagnosis of MetS^25^. CT is the gold standard measurement method for visceral fat is CT. However, previous studies on MetS prediction have been conducted without obtaining data on visceral fat^12–23^ because of the time-consuming and invasive process . We built a device that measures visceral fat non-invasively and constructed human dataset that includes VFA between 2015–2020. We investigated the relationship between MetS onset and visceral fat, and developed a MetS onset prediction algorithm. The dataset comprised 169 and 1058 cases in the MetS onset and non-onset groups, respectively. The percentage of cases affected by visceral fat onset within three years was 13.8%. Analysis of the baseline VFA and MetS onset using a box plot showed that the VFA quartiles in the onset group were 25%: 92 cm^2^; and 75%, 130 cm^2^, and the VFA quartiles in the non-onset group were 25%: 42 cm^2^; and 75%, 85 cm^2^; showing that the data overlap was quite small (Fig. 2A). These results indicated a large difference in the initial visceral fat amount between the onset and non-onset cases. The ROC AUC of 0.8597 ± 0.0241 (95% Confidence Interval) suggested that the baseline visceral fat was a strong factor influencing the prediction of MetS onset. The MetS cut-off value was found to be 82.5 cm^2^. Many cross-sectional studies have reported that visceral fat is associated with MetS onset^50,51^. Regarding cut-off values of VFA for MetS, a cross-sectional study of type II diabetes patients aged 18–75 years in China reported values of 84.7 cm^2^ in males and 81.1 cm^2^ in females^32^. A cross-sectional study of Chinese patients aged 35–75 years reported cut-off values of 79.2 cm^2^ for both males and females^30^. A longitudinal study reported values of 84 cm^2^ and 58 cm^2^ in Korean males and females, respectively^36^. In the present study, the MetS cut-off value was 82.5 cm2 in the database, which included both men and women (Fig. 2B), and the above previous studies suggest that our study was valid. Visceral fat area is known to be a useful indicator for assessing the accumulation of obesity-related cardiovascular risk factors^52–55^, while MetS is recognised worldwide as a useful indicator for predicting cardiovascular risk^56^. Currently in Japan, 100 cm2 is the cut-off value for metabolic syndrome for both men and women^25,26^, but the cut-off leading to future onset of metabolic syndrome is 82.5 cm2, which should be the proposed cut-off value for early prevention.

Our established non-invasive device for the non-invasive measurement of visceral fat will enable more extensive screening and monitoring of visceral fat levels. The device may therefore facilitate early detection of individuals at risk of MetS, enable timely lifestyle interventions and reduce the burden of associated health complications. Incorporating visceral fat measurements into predictive algorithms could improve public health outcomes by providing a practical tool that can guide individuals to take preventive measures before the onset of MetS.

We built an algorithm for predicting MetS onset using visceral fat. There are multiple algorithms available worldwide for predicting MetS onset ^12–23^. One challenge in predicting MetS onset is that the diagnostic definition of MetS differs in each country. In Japan, despite the fact that visceral fat is an essential parameter, no algorithm for predicting disease onset using visceral fat values has been constructed thus far. Therefore, in this study, we developed an onset prediction algorithm using visceral fat measurements as the input parameter. As mentioned above, the relationship diagram between the risk of developing metabolic syndrome and visceral fat and the ROC AUC results suggest that visceral fat area is the most important parameter for creating a prediction model for that risk, and that visceral fat area alone is sufficient as an input parameter for the model. However, six other parameters have also been reported to be associated with metabolic syndrome, so a model including these was created. Although multiple regression is available as a learning model, machine learning was also included in this study. Examination results confirmed the successful construction of an onset prediction algorithm using six parameters in addition to visceral fat (Model 2) (AUC = 0.9004 ± 0.0316 in cross validation data (95% Confidence Interval)). On the test data, the model yielded an AUC of 0.8836 ± 0.0662 (95% Confidence Interval) (Fig. 3). Although no strict definition of overfitting exists, if there is a significant difference between the cross-validation AUC and the test AUC of the constructed predictive model, overfitting due to small data set size is considered to exist. However, in the present study, no significant differences in AUC differences were found in the VFA model with six parameters (Model 2) as well as in the VFA alone model (Model 3). Therefore, overfitting is not considered to be a concern.

The feature impact analysis showed visceral fat to be a dominant contributor (Fig. 4). There have previously been many onset prediction algorithms that use blood items^13–23^. The visceral fat meter developed in this study is a non-invasive device. The algorithm developed in this study demonstrated that MetS onset can be predicted based on non-invasively measured parameters such as visceral fat, BMI, number of cigarettes smoked, gender, age, and DBP. Although this device has previously been used for visceral fat measurements, the findings of this study may result in the expansion of the functionality of the device. The MetS algorithm uses measurements of only non-invasive parameters and has high medical interpretability; thus, it is expected to facilitate easy and convincing understanding of onset risk. Moreover, it can be used in a variety of applications. Algorithm problems often involve regional differences; however, because the developed algorithm uses parameters with high medical interpretability, there may be fewer validation tests required. Medical expenses for obese people with visceral fat are higher than that for obese people without visceral fat^46^, and the algorithm developed in this study can guide people to reduce their visceral fat before MetS onset.

In this study, the prediction model with six parameters associated with MetS (BMI, number of cigarettes smoked, gender, age, DBP, and SBP) in addition to VFA (Model 2) exhibited a high prediction accuracy (cross-validation AUC = 0.9004 ± 0.0316, test AUC = 0.8836 ± 0.0662 (95% Confidence Interval)). The prediction accuracy significantly decreased when replacing VFA with waist circumference in Model 2 (Supplemental Fig. 4). Therefore, it was inferred that VFA was a more important factor than waist circumference when predicting MetS onset. Previous research shows only a weak correlation between VFA and waist circumference (males: *r* = 0.68, females: *r* = 0.65), with considerable individual-level variance between the VFA and waist circumference readings. For example, in the Japanese population, males with a waist circumference of 85.0–86.0 cm have a VFA of 67–137 cm^2^^26^.

We obtained six years of data on visceral fat, and built a MetS onset prediction model to determine whether onset would occur within three years of the baseline time. Previous research did not measure visceral fat, and the models generally include blood data, which can be an obstacle in daily monitoring. Therefore, the present study added medically important evidence after accurately examining a prediction model with an eye toward social implementation. The present study has several limitations. As the model constructed in this study does not include genetic and environmental factors as input factors, the model may not be applicable in populations where these factors differ; thus, the model’s performance must be checked by including participants from different races and regions. The MetS onset risk prediction model is a just a guidance tool, and when implemented, people must be asked to change their lifestyles and later confirm whether there is an actual decrease in the incidence of MetS. The visceral fat area measured by a visceral fat meter is highly correlated with that measured by CT, which is the gold standard. The visceral fat meter is used as a medical device in clinical practice. As this model is a study directly confirming the relationship between the visceral fat and metabolic risk, we consider that there is a potential influence of measurement error of the visceral fat. Additionally, the model has not yet been calibrated to compare its predictions with actual outcomes. Conducting such calibration in the future would help enhance the model’s applicability and reliability across diverse populations and settings.

## Conclusions

We built a six-year medical dataset that included visceral fat measurements. Visceral fat was found to be an important factor for determining the onset of MetS in the future. We developed a high-accuracy onset prediction algorithm using non-invasive parameters, including visceral fat. Further validation of the model in diverse populations, as well as the exploration of additional predictors that may enhance the model’s performance, will be undertaken.

## Methods

### Dataset construction

The Iwaki Health Promotion Project^57^ is an annual health check-up program for residents aged 20 years or older, started in 2005 and conducted in Hirosaki City, Aomori Prefecture, Japan. A variety of data were collected in this project, including those on genes, physical characteristics, behavioural habits, and intestinal bacteria. Visceral fat data were collected from 2015 onwards, and this study used data for the period 2015–2020. The health examination data included visceral fat area (VFA), blood data, dietary data, and interview data. VFA was estimated by the abdominal bio-impedance method using EW-FA90 (Panasonic Corporation), an approved medical device in Japan (No. 22500BZX00522000)^58^. Measurement values from this device have been reported to have a strong correlation with those obtained using computed tomography (CT), which is the gold standard for measuring visceral fat^58^. Other basic blood parameters were obtained as previously reported^59–61^. We previously used the data from the present study to conduct research on diet^59,62^, daily gait speed^60^, and intestinal bacteria^61^. This study was approved by the Hirosaki University Graduate School of Medicine ethics committee (approval number: 2014–377-1, 2016–028-1, 2021–030, 2018–012, 2020–046-4, 2020–046-1) and conducted in accordance with the recommendations set out in the Declaration of Helsinki. All participants provided written informed consent.

### MetS onset criteria

The following were used as MetS diagnosis criteria. The presence or absence of MetS was determined for each participant at each time point in the dataset^25^.

Required criteria: “VFA ≥ 100 cm^2^ (for both males and females)”.

One of the following conditions apply:

(1) Triglycerides (TG) ≥ 150 mg/dL, high-density lipoprotein cholesterol (HDLC) < 50 mg/dL, orcurrently receiving medication for that disease; or all three.

(2) Systolic blood pressure (SBP) ≥ 130 mmHg, diastolic blood pressure (DBP) ≥ 85 mmHg, or taking antihypertensive medication; or all three.

(3) Blood sugar (BS) ≥ 110 mg/dL, receiving antidiabetic therapy, or both.

Those who was not diagnosed with metabolic syndrome at all during the 3 years were labeled as the non-onset group, and those who diagnosed with the syndrome after 1 to 3 years were labeled as the onset group. Finally, from the data compiled from six years of Iwaki Health Promotion Project (n = 5,905), we excluded those participants who were not assigned a label within three years (n = 3,870) and those who currently fell under the category of MetS (n = 502) to create the final dataset (n = 1,533).

### Data pre-processing

We used the DataRobot AI Platform (ver.9.1, DataRobot, Supplemental Method 1) to convert the numerical and categorical variables into the format that was most appropriate for each model, including standardisation, missing value imputation, one-hot encoding, rigid transformation, and ordinal encoding.

### Dataset creation and analysis method

The dataset (*n* = 1533) was randomly divided into training data for five-fold cross-validation (80%, *n* = 1227) and test data (20%, *n* = 306) using DataRobot (Fig. 1). To ensure data integrity and prevent leakage, we used “group” partition and set “individual ID” as the group ID when dividing the data. This method ensures that all data points associated with a specific individual remain within the same data subset (either training, validation, or test data). The rationale for using the group partitioning method is to prevent data leakage by ensuring that data from the same individual (or lot) is not spread across training, validation, and test data. This method maintains data integrity and independence between these sets, which is crucial for accurately evaluating model performance on unseen data. We computed the accuracy and stability of each model using five-fold cross-validation within the training data to select the optimal model. Subsequently, the test data, which was entirely independent from the model creation process, was used to estimate model prediction accuracy for unknown data.

The data for cross validation were used to confirm the effect of visceral fat on MetS onset. Differences between each parameter of the MetS onset and non-onset groups were analysed using R software version 4.3.1, and Fisher’s exact and Mann–Whitney U (corrected for ties) tests were applied for the categorical and continuous variables, respectively.

We evaluated the effect of visceral fat on the onset of MetS as follows. We used box plots, the exact Mann–Whitney U test, and logistic regression analysis adjustments for confounding factors (gender, age, number of cigarettes smoked, alcohol intake, and exercise intensity) to obtain receiver operating characteristic (ROC) curves using R and calculated the VFA cut-off value for MetS onset risk based on the Youden index^63^ or the ROC curve closest to (0,1)^64,65^.

Next, we built an MetS onset prediction model using the cross-validation data. First, we calculated the area under the curve (AUC), using VFA as the only parameter. We then considered seven additional parameters previously reported to be associated with MetS (gender, age, BMI, systolic blood pressure, diastolic blood pressure, number of drinking days per week, and number of cigarettes smoked)^12^ for a total of eight input features in the prediction model. Machine learning was employed using DataRobot to estimate their effect on prediction accuracy. The model with the highest prediction accuracy based on the AUC score was chosen from the 18 models that come standard with DataRobot. The chosen model was used for MetS onset risk predictions on the test dataset and calculate the AUC score. The 18 models included general linear and classification models, including XGBoost, Elastic-Net, LightGBM, logistic regression, and residual neural networks. DataRobot automatically tunes the hyperparameters of models, Supplemental Fig. 1, 2 describe the hyperparameters. We used an AUC difference test (DeLong’s test^66^) for the AUC calculated from this analysis. For each model, we analysed the difference between the cross-validation and test AUCs. We visualised the contribution of each of the eight features to the MetS onset risk using the SHapley Additive exPlanations (SHAP)^67^ function of DataRobot. First, we used SHAP to visualise the effect of fluctuations in a given feature on the onset risk of each individual. Next, we calculated the absolute value of the degree of influence of each feature in each individual. The relative magnitude of the averaged value was compared and displayed as the feature impact. This facilitated ranking the features according to their contribution to MetS onset risk.

## Supplementary Information

Supplementary Information.

## Data Availability

The data cannot be shared publicly because of ethical concerns. Data are available from the Hirosaki University COI Institutional Data Access/Ethics Committee (contact via e-mail: coi@hirosaki-u.ac.jp) for researchers who meet the criteria for data access. Researchers need to be approved by the research ethics review board of the organisation of their affiliations.
