# Correction: Effect of visceral fat on onset of metabolic syndrome

**DOI:** 10.1038/s41598-025-20722-2

**Published:** 2025-10-06

**Authors:** Hiroto Bushita, Naoki Ozato, Kenta Mori, Hiromitsu Kawada, Yoshihisa Katsuragi, Noriko Osaki, Tatsuya Mikami, Ken Itoh, Koichi Murashita, Shigeyuki Nakaji, Yoshinori Tamada

**Affiliations:** 1 Department of Medical Data Intelligence, Research Center for Health-Medical Data Science, Hirosaki University Graduate School of Medicine, Aomori, Japan; 2 Human Health Care Products Research Laboratories, Kao Corporation, Tokyo, Japan; 3 Department of Active Life Promotion Sciences, Hirosaki University Graduate School of Medicine, Aomori, Japan; 4 Research and Development, Kao Corporation, Tokyo, Japan; 5 Department of Preemptive Medicine, Innovation Center for Health Promotion, Hirosaki University Graduate School of Medicine, Aomori, Japan; 6 Department of Stress Response Science, Biomedical Research Center, Hirosaki University Graduate School of Medicine, Aomori, Japan; 7 Research Institute of Health Innovation, Hirosaki University, Aomori, Japan; 8 Department of Social Medicine, Hirosaki University Graduate School of Medicine, Aomori, Japan

Correction to: *Scientific Reports* 10.1038/s41598-025-01389-1, published online 30 May 2025

The original version of this Article contained errors.

The X-axis labels in Figure 2B, Figure 3, Supplemental Figure 3 and Supplemental Figure 4 were incorrectly given as ‘False negative rate [%]’.

The original Figure 2 and Figure 3 and accompanying legend appear below.

In addition, in the Methods section, under the subheading ‘Dataset construction’,

“The Iwaki Health Promotion Project^57^ is an annual health check-up program for residents aged 20 years, started in 2005 and conducted in Hirosaki City, Aomori Prefecture, Japan.”

now reads:

“The Iwaki Health Promotion Project^57^ is an annual health check-up program for residents aged 20 years or older, started in 2005 and conducted in Hirosaki City, Aomori Prefecture, Japan.”

The original Article and accompanying Supplementary Information file have been corrected.

**Fig. 2:**
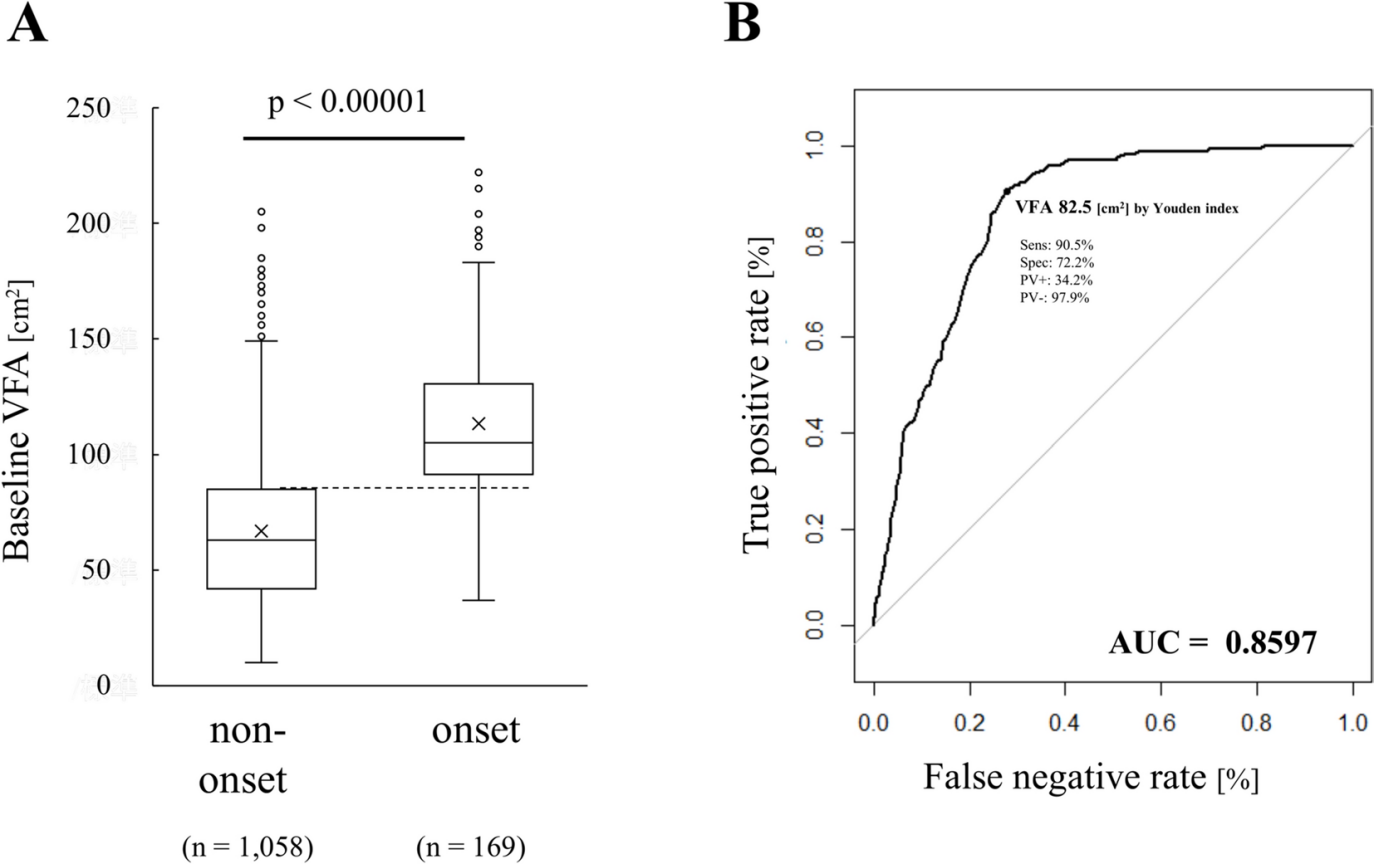
Effect of baseline visceral fat on MetS onset (**a**) VFA values at baseline in the MetS onset group (*n* = 169) and non-onset group (*n* = 1058), The p value indicates the difference between the MetS onset and non-onset groups using the Mann–Whitney U test (corrected for ties); (**b**) ROC curve of baseline VFA that determines MetS onset risk. The cut-off value was calculated using the Youden index.

**Fig. 3:**
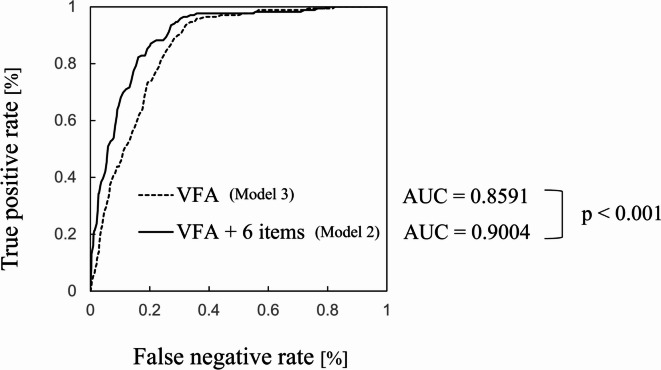
Comparison of AUC during cross-validation of the two models for MetS onset prediction. Model 3 was constructed using Elastic-Net, and the input feature was VFA. Model 2 was constructed using LightGBM, and the input features were VFA, BMI, number of cigarettes smoked, sex, age, SBP, and DBP. The AUC difference was calculated using the DeLong’s test.

## Supplementary Information

Supplementary Material 1

