# Supplementary material for: Effect of visceral fat on onset of metabolic syndrome
Source: Sci Rep. 2025 May 30;15:19012. doi: 10.1038/s41598-025-01389-1 (PMC12125335; doi:10.1038/s41598-025-01389-1)
Supplement: Supplementary file 1 — Supplementary Information. [file 41598_2025_1389_MOESM1_ESM.docx]

**Supplementary Information**

**Effect of visceral fat on onset of metabolic syndrome**

Hiroto Bushita^1,2,3^, Naoki Ozato^2,3^, Kenta Mori^2,3^, Hiromitsu Kawada^2^, Yoshihisa Katsuragi^3,4^, Noriko Osaki^2^, Tatsuya Mikami^5^, Ken Itoh^6^, Koichi Murashita^7^, Shigeyuki Nakaji^8^ and Yoshinori Tamada^1^.

^1^Department of Medical Data Intelligence, Research Center for Health-Medical Data Science, Hirosaki University Graduate School of Medicine, Aomori, Japan.

^2^ Human Health Care Products Research Laboratories, Kao Corporation, Tokyo, Japan.

^3^Department of Active Life Promotion Sciences, Hirosaki University Graduate School of Medicine, Aomori, Japan.

^4^Research and Development, Kao Corporation, Tokyo, Japan.

^5^Department of Preemptive Medicine, Innovation Center for Health Promotion, Hirosaki University Graduate School of Medicine, Aomori, Japan.

^6^Department of Stress Response Science, Biomedical Research Center, Hirosaki University Graduate School of Medicine, Aomori, Japan.

^7^Research Institute of Health Innovation, Hirosaki University, Aomori, Japan.

^8^Department of Social Medicine, Hirosaki University Graduate School of Medicine, Aomori, Japan.

Corresponding author: Yoshinori Tamada^1^

Supplemental Method 1. Model construction using DataRobot

DataRobot is an AI platform for efficient data analysis and machine learning. It incorporates the data analysis know-how of data scientists who have achieved high results in Kaggle, where data scientists from all over the world gather to compete in analytical skills. It can automate a series of processes from data pre-processing to model building, evaluation and prediction, without the need for specialist knowledge. The main processes to be automated are.

- Data pre-processing: automatic data cleaning and feature selection.

- Model building: optimal predictive models are automatically generated using multiple machine learning algorithms.

- Evaluating models: automatically evaluates the accuracy and performance of the generated models.

- Running predictions: makes predictions on new data using the built models.

- Operate and monitor models: monitor the performance of the models and automatically re-train them if necessary.


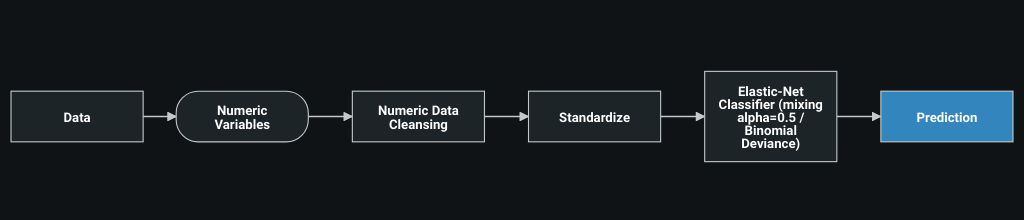
Supplemental Figure 1. Flowchart for constructing a MetS onset prediction model with VFA as the input feature, taken from DataRobot.

Hyperparameters for Elastic-Net: PostProcessing: wordcloud : False; PreProcessing: frozen parameters lid : None; Forest: n_jobs : 1; PostProcessing: stack_margin : False; PostProcessing: stack_folds : 5; fit_intercept : True; Forest: random_state : 1234; PostProcessing: right_censoring : None; random_state : 1234; tol : 0.0001; PostProcessing: prime_alpha_index : None; PreProcessing: language : None; Stepwise: backwards : 0; ShapFit: shap_fit : True; max_iter : 100; PostProcessing: stack_keep_top_n : 0; fit_alpha_scaler : True; enet_alpha : 0.5; enet_lambda : auto; beta_transform : id; ShapFit: shap_center : True; loss : log; PostProcessing: predictions_to_boost : False; warm_start : False; PostProcessing: move_imputed : False; Forest: n_estimators : 1; Stepwise: test_fraction : 0.25; PostProcessing: left_censoring : None; PostProcessing: stage : None; PostProcessing: stack : True; PostProcessing: stack_sequential : False; Decay: Type : None; PreProcessing: balance_weights : False; sigma : 1e-06


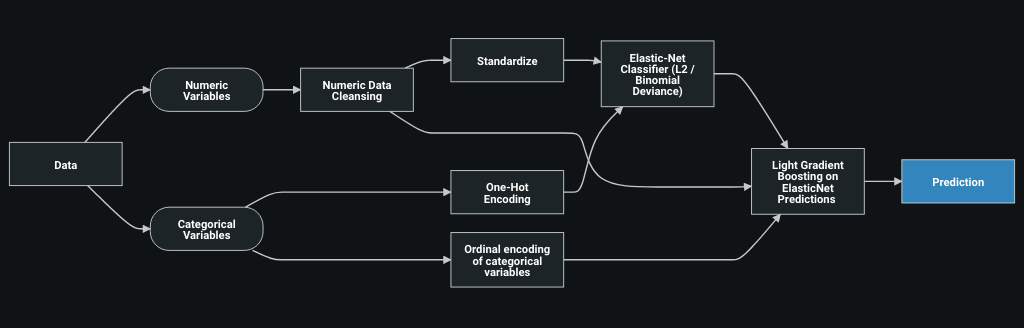
Supplemental Figure 2. Flowchart for constructing a MetS onset prediction model with VFA and six MetS-related parameters as input features taken from DataRobot.

Hyperparameters for LightGBM: n_jobs : -1; num_leaves : 2,4,16; reg_alpha : 0.0; PreProcessing: frozen parameters lid : None; early_stopping_rounds : 200; boosting_type : gbdt; PostProcessing: stack_margin : False; PostProcessing: stack_folds : 5; subsample_for_bin : 50000; min_child_samples : 10; sigmoid : 1.0; reg_lambda : 0.0; min_split_gain : 0.0; PostProcessing: right_censoring : None; Forest: random_state : 1234; max_bin : 255; objective : binary; PreProcessing: language : None; max_depth : none; Stepwise: backwards : 0; ShapFit: shap_fit : True; subsample_freq : 1; PostProcessing: stack_keep_top_n : 0; learning_rate : 0.05; PostProcessing: prime_alpha_index : None; PostProcessing: wordcloud : False; n_estimators : 1000; ShapFit: shap_center : False; Forest: n_jobs : 1; PostProcessing: predictions_to_boost : False; link_transform : None; PostProcessing: move_imputed : False; colsample_bytree : 1.0; min_child_weight : 5; Forest: n_estimators : 1; Stepwise: test_fraction : 0.25; PostProcessing: left_censoring : None; PostProcessing: stage : None; subsample : 1.0; PostProcessing: stack : True; PostProcessing: stack_sequential : False; is_unbalance : False; Decay: Type : None; PreProcessing: balance_weights : False; verbosity : 0

Supplemental Figure 3. Prediction accuracy when VFA was replaced with waist circumference as the input feature.

In both models, the learner and hyperparameters were optimised according to the input features. AUC differences between the two models were calculated using Delong’s test.

Features: VFA only, VFA; WC only, WC

Learner: VFA only, Elastic-Net; WC only, Residual Neural Network

Supplemental Figure 4. Prediction accuracy when VFA + six parameters were changed to waist circumference + six parameters.

In both models, the learner and hyperparameters were optimised according to the input features. AUC differences between the two models were calculated using Delong’s test.

Features: VFA + six parameters, VFA, BMI, SBP, Gender, DBP, Age, Cigarettes; WC + six parameters, WC, Gender, BMI, Age, SBP, DBP, Cigarettes

Learner: VFA + six parameters, LightGBM; WC + six parameters, Logistic regression

Supplemental Table 1.　Model accuracy with the input variables changed.

|  | **Model 1** | **Model 2** |  |  |  |  |  | **Model 3** |
| --- | --- | --- | --- | --- | --- | --- | --- | --- |
| **Input items** | VFA  SBP  BMI  DBP  Gender  Age  Cigarettes  Drinking | VFA  SBP  BMI  DBP  Gender  Age  Cigarettes | VFA  SBP  BMI  DBP  Gender  Age | VFA  SBP  BMI  DBP  Gender | VFA  SBP  BMI  DBP | VFA  SBP  BMI | VFA  SBP | VFA |
| **Learner** | LightGBM | LightGBM | LightGBM | LightGBM | Regularized Logistic Regression | LightGBM | Residual Neural Network | Elastic-Net |
| **AUC**  （Cross-validation） | 0.8992 | 0.9004 | 0.8974 | 0.8933 | 0.8922 | 0.8925 | 0.8938 | 0.8591 |
| **AUC**  （Test） | 0.8845 | 0.8836 | 0.8820 | 0.8837 | 0.8812 | 0.8784 | 0.8754 | 0.8686 |

Model 1 was constructed using LightGBM and input parameters VFA, BMI, number of cigarettes smoked, gender, age, SBP, DBP, and drinking firstly, and display the feature impact based on SHAP value. After excluding the lowest-ranked feature, Model 2 was constructed using LightGBM and the input parameters VFA, BMI, number of cigarettes smoked, gender, age, SBP, and DBP. The process was repeated until only one parameter remained, and Model 3 was constructed using Elastic-Net and the input parameter VFA.
